# Retraction: The Succinate Receptor GPR91 Is Involved in Pressure Overload-Induced Ventricular Hypertrophy

**DOI:** 10.1371/journal.pone.0352944

**Published:** 2026-07-02

**Authors:** The PLOS One Editors

After this article [1] was published, concerns were raised regarding overlap with a previously published article [2] by an overlapping author group that was not cited in [1], Figs 1-3 and 6-7 in [1], and compliance with the PLOS Animal Research policy. Specifically:

- Both articles [1,2] investigate the role of succinate-GPR91 signaling in a pulmonary arterial banding (PAB) model of right ventricular hypertrophy (RVH.) [1] and [2] use similar methodologies, report similar results (that succinate caused RVH in sham treated animals and accelerated RVH in PAB treated animals, and that the P13K antagonist wortmannin inhibited succinate induced RVH) and present the same conclusions (that succinate-GPR91 and the PI3K.Akt signaling pathway is involved with RVH).
- Figs 1-3 contain several graphs annotated with an undefined ‘&’ symbol.
- There does not appear to be a Fig 1A.
- Figs 6-7 contain unlabeled panels.
- The Methods do not report an ethics approval number. Additionally, the use of chloral hydrate for surgical anesthesia and euthanasia may not fully align with internationally accepted standards at the time of publication.

First author DY stated that whilst [1] and [2] reach similar conclusions, they have different scopes, methodologies and findings. Specifically, DY stated:

- In the pulmonary arterial hypertension (PAH) model, [2] describes right ventricular hypertrophy (RVH), while [1] describes right ventricular hypertrophy combined with left ventricular hypertrophy (LVH).
- [1] presents echocardiographic data of the right ventricle (RV) and left ventricle (LV), Masson trichrome staining for fibrosis assessment, and siRNA-mediated GPR91 knockdown.
- [1] includes VEGF as a marker and explores additional mechanistic pathways beyond what was reported in [2].
- [1] includes validation in human right atrial tissue samples with corresponding echocardiographic data, to provide clinical relevance absent in [2].

First authors DY and LY also stated that the animal model in [1] was established via monocrotaline (MCT) injection, rather than PAB, that references to PAB in [1] are descriptive errors, and that therefore, [1] and [2] use different animal models.

First author DY stated that the animal experiments were approved by the Ethics Committee of Nanjing Medical University under approval number IACUC-1201602. First author DY further stated that the undefined ‘&’ symbol in Figs 1-3 denotes that P < 0.05 compared to the PAB+Succinate group. First authors DY and LY provided the missing panels for Fig 1A and two differing sets of original underlying quantitative data, however, editorial assessment of these images and data raised further concerns that call into question the reliability of these results.

A member of the Editorial Board reviewed articles [1] and [2], and the author’s responses. They stated that [1] and [2] appear highly similar given that the stress is acute outflow obstruction and compensatory overgrowth of the cardiac chambers, the target is GPR91, and the analyses post injury are highly similar in [1] and [2]. They also stated that [1] contains minor additions compared to [2], including echo studies for tracking real-time changes, VEGF tracking, tissue matrix changes, and siRNA mediated GPR91 knockdown, and that whilst the latter adds some value, the remainder do not significantly extend the findings of [1] compared to [2]. The member of the Editorial Board also raised the following concerns with [1]:

- The Methods do not appear to describe details of animal treatment assignment randomization, age-matching, animal sexes, rat strains, blinding of researchers to treatments, physiology control (temperature, BP, pulse, oxygenation) under anesthesia, and post procedure pain relief.
- A power analysis does not appear to have been given for the cohort size.
- Some data have numerous treatment groups, and the sample sizes may not be large enough for a test of true variation.
- There does not appear to be evidence of primary antibody specificity.
- The western blots lack molecular marker lanes.
- In Fig 6, there do not appear to be tissue or cell images after the control siRNA transfection.
- Demographic and biometric information (age, sex, medication and disease(s) history, cohort size, balancing of cohort) do not appear to be reported which limits the value of the human sample data.

In light of the above concerns, the *PLOS One* Editors retract this article.

LY, DY, and XMM did not agree with retraction. RM, JZ, HH, LH, YF, SW, WZ, and NY either did not respond directly or could not be reached.
